# Dataset of the adoption of digital tools for research sharing among academics in African Varsities during the COVID-19 pandemic in 2020

**DOI:** 10.12688/f1000research.54245.3

**Published:** 2022-04-04

**Authors:** Valentine Joseph Owan, Jennifer Uzoamaka Duruamaku-Dim, Francisca Nonyelum Odigwe, Mercy Valentine Owan

**Affiliations:** 1 Department of Educational Foundations, University of Calabar, Calabar, 540271, Nigeria; 2 Department of Guidance and Counselling, University of Calabar, Calabar, 540271, Nigeria; 3 Department of Educational Management, University of Calabar, Calabar, 540271, Nigeria

**Keywords:** Africa, data, digital tools, higher education, research, research dissemination, staff adoption, varsities

## Abstract

This dataset was collected from a total of 1,977 university lecturers across 24 African countries, that were purposively targeted due to their level of exposure to scholarly publications. The dataset was collected through an online survey that was sent to respondents through email, WhatsApp, and the Association of African Universities Telegram group. The questionnaire was designed by the researchers and validated by five experts for face and content validity. The demographic information of the data was analysed and the softcopy of the data uploaded to the Mendeley database for easy retrieval after deidentification (see Data Availability statement). The associated questionnaire can be found in the extended data. In Africa, this appears to be the broadest dataset associated with academics’ perception of utilizing digital platforms for research sharing. This implies that scholars can use this dataset to quantitatively analyse the extent to which different digital tools are being utilized for research communication. Considering the current restrictions on in-person social gatherings due to COVID-19, researchers working on related studies may readily utilize this set of data, saving time and cost. A comprehensive but non-exhaustive number of 20 digital tools were assessed based on academics' awareness and current engagement with them, and the challenges they have faced using them. This offers a wide range of areas for studies to be anchored. Furthermore, researchers interested in specific digital tools can also evaluate the extent to which academic staff in African universities are aware of and willing to utilize them for research dissemination. This data will enable scholars and researchers in Africa and beyond to understand the extent to which academics in varsities are willing to adopt digital repositories for research sharing in the context of Africa.

## Introduction

According to the most recent statistics gathered in 2021, on the COVID-19 pandemic from Johns Hopkins University and the Africa Center for Disease Control, there are 874,036 active cases of COVID-19, with 18,498 total confirmed cases, 330,981 recoveries, and 524,557 deaths in Africa (Coronavirus Resource Centre, John Hopkins University, 2021). As cases are confirmed daily across the globe, the breakdown remains variable. As of May 13, 2020, every African country had been infected, and instances of COVID-19 have been reported in 213 nations and territories throughout the world; while the whole world grieved and felt the uncertainty and continual fear of losing their loved ones ( Cohut, 2020). The COVID-19 pandemic has posed a challenge to the way people and organizations go about their daily lives. As a result, there is an increasing need for individuals and organizations to make changes to their behaviour in order to eliminate the spread of the virus. Research sharing and communication occupy a central position in the world ( Odigwe *et al*., 2020), and has proliferated during the COVID-19 pandemic because of social distancing. Many academics have welcomed the use of digital platforms to share the results of intellectual property or research ( Bik & Goldstein, 2013; Bougioukas *et al*., 2020; Siedlok *et al*., 2020; Jarreau, 2015; Yammine *et al*., 2018; Zientek *et al*., 2018). Currently, the importance of internet technologies for wider dissemination of research results, visibility of researchers, quick file sharing, as well as the uploading and downloading of academic publications cannot be overstated. Furthermore, multimedia technologies have a huge potential for increasing the visibility, reputation, placement, and public value of academics and universities ( Anenene *et al.*, 2017).

Despite the importance of digital repositories in the academic world, they have been described as difficult to implement. For example, research has revealed that academics make little use of open-source academic resources ( Kodua-Ntim, 2020). Insufficient campaigning, ICT accessibility, facilities, finances, power supplies, lack of technical capabilities, institutional repository regulation, lack of resources, organizational culture/politics, and patent issues have all been identified as major barriers to academic personnel adopting open-access university libraries ( Kodua-Ntim, 2020; Owan *et al*., 2021). This dataset was created because, during the peak of the COVID-19 outbreak, many conferences and other intellectual gatherings were moved to internet platforms to maintain social distance and prevent the virus from spreading. The readiness of academics to accept and use online technologies may be a determining factor in the utilisation of digital platforms for such academic goals. It is critical to know how ready researchers are to use online resources for research communication, particularly from the perspective of developing African countries. This is because their level of readiness to adopt digital tools may have an impact on the utilization of digital platforms for various purposes, which in turn may have an impact on their research dissemination practices.

**Figure 1.**
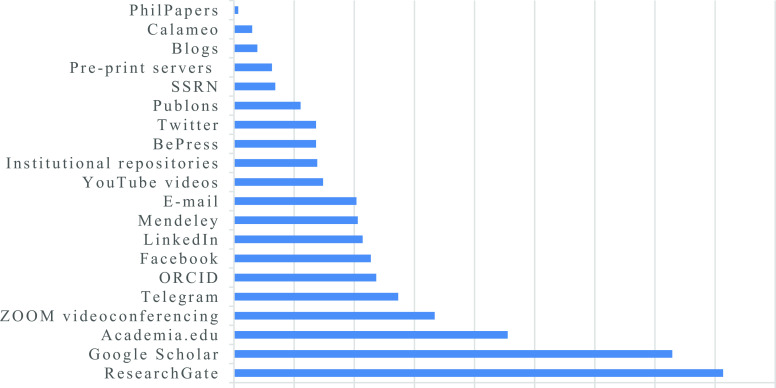
Percentage indicating the extent to which academic staff are utilizing specific digital tools for research dissemination.

This dataset is an excel document showing a person-by-item matrix of the responses to the various aspects of the online questionnaire ( Owan, 2021). There are a total of 1977 rows (representing the number of respondents) and 32 columns (representing the number of variables) in the dataset. There is one dummy variable (gender), four ordinal variables (age, educational qualification, rank, and years of work experience), and two nominal variables (research area and country of residence). Columns H to Column AA contain data on the extent staff are willing to adopt 20 unique digital tools for research sharing (see Figure 1). Column AB contains a dichotomous response of participants regarding their perception of classical/traditional versus modern/electronic approaches to research sharing. Each cell in Column AC contains a listing of platforms that respondents indicated the extent they currently utilize them. Each cell in column AD contains the total number of publications each participant has published, while column AE contains the total number of respondents’ scholarly works that are currently on the internet. The data in column AD and AE can be used to compute the ratio of scholars' work that is on the internet as a percentage of their total number of publications. Lastly, column AF contains qualitative data on the challenges scholars face in using digital tools for research sharing.

## Methods

An electronic survey, consisting of three main parts, was used for data collection. The survey was created by the researchers, using information from a review of related literature ( Owan, ‘Electronic …’, 2021). Google forms was used in designing the survey for financial reasons and because it was easy to use. The virtual snowball approach was used in targeting the respondents using the Association of African Universities’ (AAU) Telegram platform. Some respondents were sent a link to the survey and asked to forward it to other colleagues who are academic staff. The AAU Telegram is composed of academic staff from different universities across Africa. This qualified all of them as participants for the study.

### Ethics and consent

This study involved human subjects whose consent were sought in the cover letter of the questionnaire. The authors stated clearly to the respondents what the research involved and how the data will be handled. Respondents were made to understand that by completing the survey, they have consented to participate. Ethical approval was not required for this study according to the National Health Research Ethics Committee of Nigeria guidelines (NHREC).

### Instrument development

The instrument used for data collection was designed by the researchers using information from a review of related literature. The instrument was designed in three sections. Section one included a lengthy cover letter detailing the purpose of the study, its duration, the planned delivery period, the nature of the responses/data needed and an ethical declaration stating how privacy and confidentiality would be maintained. The respondents were assured that all the information solicited was going to be used for the research and that no personal data was going to be revealed to anyone. The decision to participate was voluntary and respondents were further assured that collected data would be aggregated with all identifying information removed. At the end of the letter in section 1, respondents were informed that responding to the items in subsequent sections of the questionnaire, would indicate that they have consented to participate. Section 2 gathered demographic information from respondents, such as gender, age, qualifications, academic ranks, years of job experience, areas of research and countries of residence. The demographic information was gathered using a list of options for respondents to tick.

There was no potential bias, since the research was exploratory and the medium used for data collection was transparent, inclusive and open to all African universities. The researchers had no control of snowballing distribution to subsequent participants.

The third section was divided into six sub-sections. The first sub-section was a five-point rating system for a set of 20 online sites in which participants were supposed to indicate their willingness to use them for research sharing (See Table 1). The scale response options ranged from zero (No willingness) to five (very willing). Table 1 provides information about the extent of staff willingness to utilise digital tools for research dissemination using mean and standard deviation. The grand mean of 5839 was derived from finding the average of all the mean values for the 20 specific sites. This values indicates the overall extent respondents are willing to utilise digital tools for research dissemination. The respective mean values for the 20 digital tools indicates on average, the extent to which specific platforms are desired for utilisation. The second sub-section consisted of a closed-ended inquiry designed to ascertain academic personnel perceptions of conventional and contemporary approaches to research sharing. The third sub-section was a checklist of 20 websites on which respondents could check the ones they currently use for research sharing. We visualised the resources scholars are interested in using and identified the resources that are more likely to be utilised in the future ( Figure 1). Table 2 provides information on the current state of academic staff utilisation of myriad digital tools in the dissemination of research.

**Table 1.** Extent of respondents’ willingness to adopt digital tools for research dissemination in Africa (N = 1,977).

S/N	Digital tools	Score	$\bar{X}$	SD	S ^2^	Remark
1	ResearchGate	8814	4.46	.89	.79	High extent of willingness
2	Google Scholar	8901	4.50	.78	.61	High extent of willingness
3	Publons	4860	2.46	1.97	3.88	Low extent of willingness
4	ORCID	6027	3.05	2.01	4.05	High extent of willingness
5	Mendeley	6570	3.32	1.91	3.66	High extent of willingness
6	SSRN	4680	2.37	2.01	4.05	Low extent of willingness
7	Academia.edu	7290	3.69	1.54	2.38	High extent of willingness
8	Calameo	3870	1.96	1.72	2.95	Low extent of willingness
9	BePress	3960	2.00	1.76	3.09	Low extent of willingness
10	Facebook	5670	2.87	1.98	3.93	High extent of willingness
11	Twitter	4860	2.46	1.75	3.06	Low extent of willingness
12	LinkedIn	5670	2.87	1.79	3.20	High extent of willingness
13	Telegram	5940	3.00	1.53	2.35	High extent of willingness
14	Blogs	5220	2.64	1.82	3.32	High extent of willingness
15	YouTube videos	5940	3.00	1.88	3.54	High extent of willingness
16	E-mail	8091	4.09	1.16	1.36	High extent of willingness
17	PhilPapers	3960	2.00	1.73	3.00	Low extent of willingness
18	Institutional repositories	6840	3.46	1.72	2.96	High extent of willingness
19	ZOOM video conferencing	7020	3.55	1.90	3.60	High extent of willingness
20	Pre-print servers	5580	2.82	1.97	3.87	High extent of willingness
	*Average*	*5839*	*3.03*	*1.69*	*2.98*	*High extent of willingness*

**Table 2.** Digital tools utilized by academic staff for research dissemination and the rate of utilization (N = 1,977).

S/N	Digital tools	Frequency (f)	Rate (%)
1	ResearchGate	1608	81.34
2	Google Scholar	1441	72.89
3	Academia.edu	900	45.52
4	ZOOM video conferencing	660	33.38
5	Telegram	540	27.31
6	ORCID	468	23.67
7	Facebook	450	22.76
8	LinkedIn	423	21.40
9	Mendeley	407	20.59
10	E-mail	403	20.38
11	YouTube videos	293	14.82
12	Institutional repositories	274	13.86
13	BePress	270	13.66
14	Twitter	270	13.66
15	Publons	219	11.08
16	SSRN	136	6.88
17	Pre-print servers	125	6.32
18	Blogs	77	3.89
19	Calameo	60	3.03
20	PhilPapers	14	0.71

**Figure 2.**
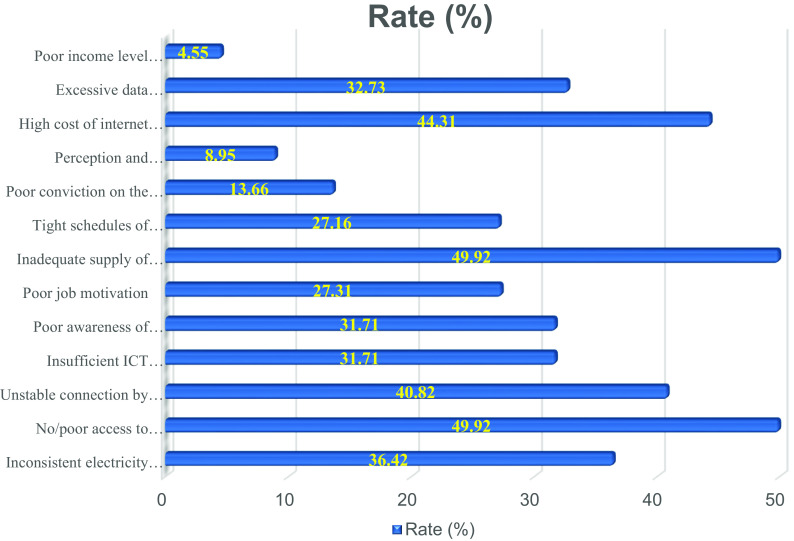
Bar chart showing the challenges faced by academics in the utilization of digital tools for research dissemination in African Universities.

The fourth sub-section was structured to assess respondents’ total number of scholarly publications (including journal articles, theses/dissertations, conference papers, book chapters, and books). Textboxes were provided for the respondents to write the total number of their published works. The fifth sub-section focused on the overall number of respondents’ academic publications available online (including those on the websites of publishers and those that are manually submitted to internet sites). The sixth sub-section was created to allow respondents to share their thoughts on the difficulties they experience while attempting to use online channels for research distribution. The bar chart in Figure 2 highlights the challenges limiting African scholars’ use of online for research dissemination. The various tools were chosen for each sub-section because of their effectiveness in collecting the required information.

For face and content validity, the survey was reviewed by three instructional technology specialists and two psychometrists from the University of Calabar's Faculty of Education. The face and content validity were carried out to ensure that the contents and arrangements of the questions/items were reasonably clear, eliminating extraneous information and ambiguity.

A pretesting pilot study ( Baker, 1994) was implemented in the study to try-out the instrument for data collection. In the pilot test a total of 60 academic staff from six countries in Africa were selected purposively from the AAU Telegram group to take part in the trial test. These 60 respondents were excluded from participating in the main study. The 60 participants took the survey once. Thereafter, 10 respondents of the 60 who participated in the pilot study were further selected to participate in a focus group discussion (FGD). The FGD, which took place on ZOOM videoconferencing, gave participants the opportunity to qualitatively discuss their experiences with the survey in terms of the length of the survey, time taken to complete it, how well they thought items measured their targeted variables and if there was an omission of anything important. Using the feedback from the focus group discussion, some online repositories were replaced. For instance, the ERIC and ProQuest databases were initially listed, but they were replaced with SSRN and Philpapers respectively, because the participants felt they cannot post documents to the previous two easily. The databases used for the replacements were suggested by the participants.

For reliability, the Cronbach alpha method was used to ascertain the degree of internal consistency of the instrument. The responses of the 60 participants in the pilot study was used. Reliability analysis was only performed for one variable (staff willingness to adopt digital tools) because it was the only variable that received continuous data. As a proof that the instrument was highly reliable, an alpha coefficient (α = 0.894) was obtained. This value was considered high as per the recommendations of other researchers (e.g., Cho & Kim, 2015; Hoekstra *et al.*, 2019; Spiliotopoulou, 2009; Taber, 2018).

The link to the e-survey was shared on the Telegram forum of the Association of African Universities, which has 1,622 participants from various African countries and regions. Members of the group, who were university academic employees, were invited to complete the survey and post it on their institutions' internet-based forums. The researchers warned the participants intending to share the link to the questionnaire, to distribute it to only academics who meet the selection criteria. To be qualified as a respondent for the study, an academic staff must have obtained a doctorate degree and must have published an article in a peer reviewed journal. Through further dissemination of the questionnaire by the initially targeted participants, the researchers were able to get information from 1,977 respondents. All the respondents provided complete information to the questionnaire items; thus, there was no missing data.

The survey was open from July 2020 to January 2021, reflecting a seven-month data collection period. The accompanying data was imported, translated to an Excel file (.xlsx), reviewed, cleaned, and re-coded after the survey was closed (where necessary). The data obtained was deidentified in line with the privacy statement given to participants following the Safe Harbour method. All information pertaining to dates such as age and years of experience, were grouped into a range (e.g., 20 – 29 years). The data was cleaned in sections where respondents were given the freedom to provide short answers. Cleaning was done through fixing spelling errors, matching cases for responses and grouping responses. The survey received feedback from 1,977 academics in African universities. The survey was stopped not because of saturation but because no further response was obtained two months after the last response was obtained. At this point it became clear that responses were no longer forthcoming.

## Data availability

### Underlying data

Mendeley: Electronic Cross-Sectional Data of Academic Staff Preparedness to Adopt Digital Tools for Research Sharing in African Varsities. http://dx.doi.org/10.17632/69k939yr4n.4 ( Owan, ‘Electronic …’, 2021).

The project contains the following underlying data:

- Utilization of Digital tools for Research Sharing Questionnaire.pdf
- Utilization of Digital Tools for Research Sharing Questionnaire Responses.csv
- Utilization of Digital Tools for Research Sharing Questionnaire Responses-8eV2iY.xlsx
- Utilisation of Digital Tools for Research Sharing Responses .sav
- Utilisation of Digital Tools for research Sharing further analysis.xlsx
- Utilisation of Digital Tools for Research Sharing further analysis 2.xlsx

### Extended data

This project contains the following extended data:

- Utilization of Online Platforms for Research Dissemination Questionnaire (UOPRDQ)

Data are available under the terms of the Creative Commons Attribution 4.0 International license (CC-BY 4.0).
