# Supplementary material for: Dataset of the adoption of digital tools for research sharing among academics in African Varsities during the COVID-19 pandemic in 2020
Source: F1000Res. 2022 Apr 4;11:82. Originally published 2022 Jan 24. [Version 3] doi: 10.12688/f1000research.54245.3 (PMC9046901; doi:10.12688/f1000research.54245.3)
Supplement: Peer review history

# Peer review: Reviewer response for version 3

**DOI:** 10.5256/f1000research.123027.r129800

**Published:** 2022-04-27

**Authors:** Abdullah Noorhidawati

**Affiliations:** 1 Department of Library and Information Science, Faculty of Arts and Social Sciences, Universiti Malaya, Kuala Lumpur, Malaysia

The authors have made the requisite revisions. I approved the revision. The article is ready for indexing.

Are sufficient details of methods and materials provided to allow replication by others?

Partly

Is the rationale for creating the dataset(s) clearly described?

Yes

Are the datasets clearly presented in a useable and accessible format?

Yes

Are the protocols appropriate and is the work technically sound?

Yes

Reviewer Expertise:

NA

I confirm that I have read this submission and believe that I have an appropriate level of expertise to confirm that it is of an acceptable scientific standard.

# Peer review: Reviewer response for version 3

**DOI:** 10.5256/f1000research.123027.r126004

**Published:** 2022-04-14

**Authors:** Neha Lata

**Affiliations:** 1 Department of Library and Information Science, Babasaheb Bhimrao Ambedkar University, Lucknow, Uttar Pradesh, India

Having gone through the article once again, I think the authors have incorporated the suggestions of my co-reviewer adequately. This gives the article a much better look.

Are sufficient details of methods and materials provided to allow replication by others?

Yes

Is the rationale for creating the dataset(s) clearly described?

Yes

Are the datasets clearly presented in a useable and accessible format?

Yes

Are the protocols appropriate and is the work technically sound?

Yes

Reviewer Expertise:

Management of Electronic Resources, ICT applications to Library and Information Services, Digital Library and Information Retrieval System, Library Automation and Networking

I confirm that I have read this submission and believe that I have an appropriate level of expertise to confirm that it is of an acceptable scientific standard.

# Peer review: Reviewer response for version 2

**DOI:** 10.5256/f1000research.121855.r126003

**Published:** 2022-03-28

**Authors:** Abdullah Noorhidawati

**Affiliations:** 1 Department of Library and Information Science, Faculty of Arts and Social Sciences, Universiti Malaya, Kuala Lumpur, Malaysia

I am satisfied with most of the authors’ revisions, but there are still a few things that could be improved upon/clarified:

 Firstly, I would still maintain that there are 1977 **rows **(representing the number of respondents) and 32 **columns** (representing the number of variables) in the dataset - (note to author - based off of the excel form provided).

 Secondly, I would recommend changing the term to the **digital tool** at these occurrences to make it consistent throughout the article (recommendations in bold)

- Utilisation of **online platforms** for research dissemination further analysis 2.xlsx
- Utilisation of **online platforms** for research dissemination further analysis.xlsx
- Utilisation of **online platforms** for research disseminato=.sav
- Utilization of **Online Platforms** for Research Dissemination Questionnaire (UOPRDQ) (Responses).xlsx
-  Utilization of **Online Platforms** for Research Dissemination Questionnaire (UOPRDQ)

Are sufficient details of methods and materials provided to allow replication by others?

Partly

Is the rationale for creating the dataset(s) clearly described?

Yes

Are the datasets clearly presented in a useable and accessible format?

Yes

Are the protocols appropriate and is the work technically sound?

Yes

Reviewer Expertise:

NA

I confirm that I have read this submission and believe that I have an appropriate level of expertise to confirm that it is of an acceptable scientific standard, however I have significant reservations, as outlined above.

# Response

**Published:** 2022-03-28

**Authors:** Valentine Owan

**Affiliations:** University of Calabar, Nigeria

Thank you reviewer for these two suggestions. We totally agree with you this time regarding the 1977 rows and 32 columns. Secondly, we have renamed the files in the project for consistency. We think the article will benefit from one last minor revision.

# Peer review: Reviewer response for version 1

**DOI:** 10.5256/f1000research.57710.r121430

**Published:** 2022-02-25

**Authors:** Abdullah Noorhidawati

**Affiliations:** 1 Department of Library and Information Science, Faculty of Arts and Social Sciences, Universiti Malaya, Kuala Lumpur, Malaysia

The abstract provides clear information.

 The introduction is good and cited recent references. But please check again the following statement: *“There are a total of 32 rows and 1,977 columns, with the first row indicating the variables.” *It should be 1,977 rows (indicating number of respondents) and 32 columns (the variables).

 Figure 1 should include label for the data on the bar chart, either percentage or frequency. Table 1 and Table 2 should include the N (number of responses).

*Method*

 In the abstract it was mentioned - “ *This dataset was collected from a total of 1,977 university lecturers across 24 African countries”. *But in the Introduction it was reported - ” *The link to the e-survey was shared on the Telegram forum of the Association of African Universities, which has 1,622 participants from various African countries and regions.” *Please check again the figure, as the responses gathered (1,977) were more that the total Telegram participants (1,622).

 The method section is quite clear, but I have some comments for improvement:

- It would be good to have a separate sub-section on instrument development, where information on each section of the survey instrument is discussed, currently it is all under Ethics and Consent sub-section.
- It is a good practice to include Ethics and Consent sub-section.
- Include information on the scale used to measure “willingness” as shown in Table 1.
- Provide explanation on the Average Score = *5839* as indicated in Table 1.
- Report on the pilot study and the changes made after it was conducted.
- A total of 1,9777 responses were gathered, all of them are usable data/completed responses? It is good to report the incomplete survey (that was excluded from the analysis) if that is the case. If none please inform so.

The title used the term “digital tools” but throughout the article various term were used such as internet platform, digital platform, online platform, internet-based platform – one consistent term should be used throughout the article and survey instrument to avoid confusion.

Are sufficient details of methods and materials provided to allow replication by others?

Partly

Is the rationale for creating the dataset(s) clearly described?

Yes

Are the datasets clearly presented in a useable and accessible format?

Yes

Are the protocols appropriate and is the work technically sound?

Yes

Reviewer Expertise:

NA

I confirm that I have read this submission and believe that I have an appropriate level of expertise to confirm that it is of an acceptable scientific standard, however I have significant reservations, as outlined above.

# Response

**Published:** 2022-02-25

**Authors:** Valentine Owan

**Affiliations:** University of Calabar, Nigeria

**Response to reviewer**

**Comment #1**: The abstract provides clear information.

**Response#1**: Thanks

***Comment #2: *** The introduction is good and cited recent references. But please check again the following statement: “There are a total of 32 rows and 1,977 columns, with the first row indicating the variables.” It should be 1,977 rows (indicating number of respondents) and 32 columns (the variables).

***Response #2:***  The researchers are grateful to the reviewer for the inputs. However, we tend to disagree with the reviewer regarding the statement made about rows and columns. There are 1,977 columns (representing the number of respondents) and 32 rows (representing the number of variables). Columns are vertically arranged, while rows flow horizontally. The reviewer should kindly recheck that. However, we have modified the statement to make it clearer.

***Comment #3: *** Figure 1 should include a label for the data on the bar chart, either percentage or frequency. Table 1 and Table 2 should include the N (number of responses).

**Response #3**: We are grateful to the reviewer for this insightful observation. We have indicated the percentage for Figure 1 for clarity. We have also provided the number of responses (N) to the titles of Tables 1 and 2.

*Method*

***Comment #4:*** In the abstract, it was mentioned - “This dataset was collected from a total of 1,977 university lecturers across 24 African countries”. But in the Introduction, it was reported -” The link to the e-survey was shared on the Telegram forum of the Association of African Universities, which has 1,622 participants from various African countries and regions.” Please check again the figure, as the responses gathered (1,977) were more than the total Telegram participants (1,622).

***Response #4:*** We thank the reviewer for bringing this point out. However, we are clarifying that the virtual snowball was used. Although there were 1,622 members in the Telegram group, the respondents further distributed the link to the questionnaire to other institution-specific online groups. Thus, the responses gotten was beyond the number of participants in the group. It was not an error.

***Comment #5: *** The method section is quite clear, but I have some comments for improvement: It would be good to have a separate sub-section on instrument development, where information on each section of the survey instrument is discussed, currently it is all under Ethics and Consent sub-section. It is a good practice to include Ethics and Consent sub-section.

***Response #5: *** The authors are very impressed with this comment. The original draft of the article had this section, but it was recommended for removal during the initial editorial check. Nevertheless, we have added the section “instrument development” to the revised article because we agree with the reviewer that it will make it clearer to readers. We are grateful.

***Comment #6:***   Include information on the scale used to measure “willingness” as shown in Table 1. Provide an explanation on the Average Score = 5839 as indicated in Table 1.

***Response #6: *** The researchers wish to draw the attention of the reviewer to the description of the third section of the survey, where it was stated that a five-point rating scale was used. However, in the revision, we have stated that a scale with response options ranging from 0 (no willingness) to 5 (very willing) was used.  The average score of 5839 in Table 1 has also been explained in the revised version.

    

***Comment #7:*** Report on the pilot study and the changes made after it was conducted.

***Response #7:*** Thanks to the reviewer. Although we felt it was not necessary to document the pilot study procedure in a Data Note. However, following your suggestion, we can confirm that details of the pilot study including reliability are now provided.

***Comment #8: *** A total of 1,9777 responses were gathered, all of them are usable data/completed responses? It is good to report the incomplete survey (that was excluded from the analysis) if that is the case. If none please inform so.

***Response #8: *** We are grateful to the reviewer for raising it. It is important information that should be added. As you will see in the revised version of the article, a statement has been made that complete data were collected from the 1,977 respondents without any missing data.

***Comment #9: *** The title used the term “digital tools” but throughout the article, various terms were used such as internet platform, digital platform, online platform, internet-based platform – one consistent term should be used throughout the article and survey instrument to avoid confusion.

***Response #9: *** The authors can confirm that the term "digital tools" is now consistently used in the revised version of the article.

# Peer review: Reviewer response for version 1

**DOI:** 10.5256/f1000research.57710.r120882

**Published:** 2022-02-18

**Authors:** Neha Lata

**Affiliations:** 1 Department of Library and Information Science, Babasaheb Bhimrao Ambedkar University, Lucknow, Uttar Pradesh, India

The article presents a broad dataset from 24 African countries about their intention to utilise internet-based outlets for research dissemination during the COVID-19 pandemic. The paper's general quality is good: it is well-written, and some important considerations are highlighted. The research study is well-structured, with clear ideas and concise, persuasive language. The introduction is relevant and theoretically sound. The literature evaluation is thorough, and authors successfully discussed the significance of the research from both a theoretical and practical standpoint.

 The methodology of the paper is rich and commendable because it was stated in a manner that allows for replication, and the methodologies and data analysis have no flaws in my opinion. Secondly, the authors addressed the issue of potential bias by ensuring that the link to the survey was posted only on platforms hosting academics from African universities, who in turn shared it with their colleagues.

 There was also a clear statement of ethical consideration, with evidence of a national guideline exempting the need for ethical clearance because of no associated risk of participation.

 Generally, the authors were detailed in their methodology with the safe harbour methods followed for data privacy. As a data article, the information provided is sufficient for re-use.

Are sufficient details of methods and materials provided to allow replication by others?

Yes

Is the rationale for creating the dataset(s) clearly described?

Yes

Are the datasets clearly presented in a useable and accessible format?

Yes

Are the protocols appropriate and is the work technically sound?

Yes

Reviewer Expertise:

ICT applications to Library and Information Services, Digital Library and Information Retrieval System, Library Automation and Networking, Cloud Computing.

I confirm that I have read this submission and believe that I have an appropriate level of expertise to confirm that it is of an acceptable scientific standard.
